# Supplementary material for: Are carbon emissions trading and green financial instruments synergistic? -Comprehensive quantitative research based on content analysis
Source: PLoS One. 2024 Mar 7;19(3):e0298601. doi: 10.1371/journal.pone.0298601 (PMC10919601; doi:10.1371/journal.pone.0298601)
Supplement: S1 Data — (ZIP) [file pone.0298601.s001.zip › Data/地级市Z_F工作报告.zip!/╔╜╬≈/┴┘╖┌╩╨/└┤╫╘╣1⁄2╓┌║┼í╛┬φ┐╦╩2╛▌═°í┐_2019.docx]

各位代表：

　　现在，我代表市人民政府向大会报告工作，请予审议，并请市政协委员和其他列席人员提出意见。

　　一、2018年工作回顾

　　刚刚过去的一年，是临汾经济社会发展中极其艰难的一年，也是临汾饱受涅槃之痛、奋力克难攻坚的重要一年。一年来，在省委、省政府和市委的坚强领导下，在市人大、市政协的监督支持下，市政府坚持以习近平新时代中国特色社会主义思想为指导，深入贯彻党的十九大精神和习近平总书记视察山西重要讲话精神，紧紧围绕省委“三大目标”，聚焦“345”发展战略和“12345”工作思路，全力打好防范化解重大风险、精准脱贫、污染防治“三大攻坚战”，统筹推进稳增长、促改革、调结构、惠民生、防风险，全市经济运行总体平稳，社会事业稳步发展。

　　——经济质效持续提升。地区生产总值完成1440亿元，比上年增长2.8%;全社会消费品零售总额完成687.1亿元，比上年增长7.5%;一般公共预算收入完成126亿元，比上年增长29.8%;海关进出口总额完成19.8亿元，比上年增长20.1%;规模以上工业增加值完成483.9亿元，规上工业企业利税比上年增长28.1%。

　　——产业结构不断优化。煤炭先进产能占比达到75%，全市制造业、战略性新兴产业、高新技术产业占全市工业增加值比重达到30%以上。全年实现旅游总收入634.1亿元，比上年增长31%。三次产业比重由2017年的7.2∶46.3∶46.5进一步优化为2018年的6.5∶45.9∶47.6。

　　——环境质量明显改善。市区环境空气质量综合指数为7.73，比上年下降10.1%;二级以上天数137天，比上年增加9天。六项监测指标中四项浓度值同比下降。劣Ⅴ类水体断面比例为37.5%，达到或优于Ⅲ类水体比例为25%。

　　——发展布局更加合理。不断加大布局规划和调整力度，形成了百里汾河、百里黄河、百里太岳“三个百里”功能突出、各具特色、协同发展的规划战略，并纳入了全省区域协同发展指导意见。加快产业发展布局调整，对分布在市区周边的同世达、欧环等一批焦化企业实施关闭，全面启动退城入园工作。

　　——基础设施日趋完善。持续加大城建、交通、水利等基础设施建设力度。向阳西路改造提升、锣鼓桥西延等一批城建工程按期竣工;中大街贯通、迎春街南延等部分道路完工通车;华门光影秀、汾河音乐喷泉、滨河东路道路绿化亮化等惠民工程如期完成;霍永高速西段、长临高速建成通车，环城高速全线贯通;“四好农村路”完成2287公里，尧都区、隰县被评为“四好农村路”全国示范县;沿黄旅游公路永和乾坤湾试验段全面完工;引沁入汾浮山供水工程基本完成，中部引黄、禹门口东扩涉临骨干工程扎实推进。

　　——民生事业稳步发展。城镇居民人均可支配收入完成30692元，比上年增长6.3%;农村居民人均可支配收入完成11630元，比上年增长8.3%，连续三年高于城镇居民人均可支配收入增幅。吉县、乡宁、安泽3个贫困县成功“摘帽”，隰县、蒲县、古县、浮山4个贫困县进入脱贫“摘帽”程序，全市163个贫困村退出、5.2万贫困人口脱贫，贫困发生率降至0.7%，脱贫攻坚实现了连战连胜。全市城镇新增就业和农村劳动力转移就业分别达到54911人和50917人，城镇登记失业率为2.87%。五大保险参保人数和基金征缴全部超额完成省定目标任务。34.6万农村人口稳定解决饮水安全问题。建成农村老年人日间照料中心50个。省政府六件民生实事和我市十件民生实事基本完成。

　　——政务服务全面升级。创建政务服务标准2.0体系，政务服务“一张网”建设成为全省样板;市长热线荣获全国“12345”政府服务热线“稳步攀升奖”;侯马企业投资项目承诺制“一次两会”工作法在全省推广;政府系统实现OA协同办公全覆盖;“13710”督办系统实现市、县、乡三级连通。

　　一年来，我们顶着巨大压力，在艰难中跋涉，在困境中前行，重点抓了以下几方面工作：

　　(一)抓产业、谋转型，全面加快动能转换。紧紧围绕争当“全省转型发展排头兵”的目标，把产业转型升级作为经济工作的重中之重，强力予以推进。工业方面，编制实施了《争当能源革命排头兵实施方案》，出台了煤炭、焦化、钢铁等7个行业转型升级三年行动计划，全面实施煤炭产业升级改造，加快推进焦化、钢铁产业优化布局、减量置换，强力推进铸造和装备制造、新能源、新材料、大数据等新兴产业发展。现代服务业方面，大力推进第三产业振兴行动计划，商贸物流、电子商务、文旅产业等现代服务业蓬勃发展。我市被确定为“陆港型国家物流枢纽承载城市”;兴荣物流园区智慧平台成功加盟全国物流大体系;苏宁易购入驻隰县，成为全省首家苏宁易购扶贫实体店。紧紧抓住承办山西省旅游发展大会的机遇，全力打造黄河、根祖、太行三大旅游板块，深入实施了“百项文旅活动”和“百项提升工程”，洪洞大槐树寻根祭祖园成功创建5A级景区，晋国博物馆、吉县人祖山成功创建4A级景区，克难坡等8家景区成功创建3A级景区。尧庙·华门文旅融合示范区试点起步，成功举办了早期都邑文明的发现研究与保护传承暨陶寺四十年发掘与研究国际论坛，世界大河文明国际旅游论坛会址与秘书处落户我市。现代农业方面，编制实施了《乡村振兴战略总体规划(2018—2020)》，6个县、30个乡镇、100个村试点工作扎实推进。粮食总产量达到25.7亿公斤，秋粮是历史上的第二个高产年。新发展(改造)水果8.8万亩、中药材8万亩、设施蔬菜0.5万亩、露地特色蔬菜4.2万亩。肉、蛋、奶类产量分别达34万吨、17.7万吨、6万吨。汾西洪昌养殖入选国家级龙头企业，填补了全市“空白”。

　　(二)抓改革、增活力，持续增强发展后劲。牢固树立争先意识，以时不我待的紧迫感，全面深化改革增动力，不断扩大开放激活力。扎实推进“三去一降一补”。压减煤炭产能375万吨;工业产成品存货比上年下降5.6%;商品房待售面积消化周期为5.7个月;工业企业资产负债率降低1.6个百分点;工业企业每百元主营业务收入成本降低1.3元;全市累计减免税27.3亿元，普遍性降费政策措施得到全面落实。深入推进重点领域改革。国企国资、国有林场、集体林权、金融、科技、医药卫生、财税体制等重点领域改革均取得新突破，襄汾、安泽经济技术开发区设立获批，沿黄现代农业文化旅游开发区建设在全省率先破题。大力实施创新驱动战略。深入开展高端产学研合作，与九三学社山西省委签订了《“九临合作”战略合作框架协议》，李兆申院士专家工作站落户市人民医院，李德发院士工作站落户蒲县。扎实推进“双创”活动，侯马市、华翔集团成功获批省级“双创”示范基地，隰县授渔众创空间、侯马经济技术开发区被认定为省级科技企业孵化器，临汾杏林、乡宁颐高万创等5家平台成功创建省级众创空间。“市长创新奖”持续实施，15家科技创新优秀企业获奖。全面加快对外开放步伐。加快构建内陆地区对外开放新高地，签署了《关中宣言》，加快融入关中平原城市群，与斯里兰卡阿努拉德普勒市建立了国际友好合作伙伴关系，侯马市与美国核桃市缔结为国际友好城市。创新“五大招商”模式，在北京、广州等地举办招商引资推介会10场，全年签约项目290个，总投资额1591亿元。中欧班列实现常态开行。

　　(三)抓项目、强机制，不断夯实基础支撑。按照全省“转型项目建设年”活动要求，坚持把项目工地当阵地，持续加大项目建设力度。完善项目机制。在全面贯彻落实省六项常态化机制的基础上，不断健全完善并联审批、协调调度、领导包联、项目化管理等10项工作机制，促进了项目建设的科学化、规范化。优化项目审批。加大企业投资项目承诺制改革试点工作力度，全力打造“六最”营商环境，一批项目实行了承诺制和并联审批。加快项目建设。先后四次召开高规格、大规模的工作部署会、观摩会和推进会，开展了项目集中开工月活动，全市共铺开重点项目777个，年度完成投资371亿元。推进实施产业转型项目479个，其中，新兴产业项目424个，完成投资215亿元，传统产业改造升级项目54个，完成投资42亿元。晋南钢铁集团2×1860立方产能减量置换、曲沃年产30万吨乙二醇联产LNG、山西盾安隰县风电、大宁鸿晋一次性防护手套等一批转型项目进展顺利。

　　(四)抓治理、优环境，逐步筑牢生态屏障。深入贯彻落实习近平生态文明思想，在全省率先出台《党政领导干部生态环境保护责任制实施细则》，更大力度、更高层次推进生态环境治理。认真整改突出问题。深刻汲取环保数据造假案件和三维事件教训，6个国控监测点位9大类问题及时整改完成，中央环保督察等上级督察反馈及我市自查自纠的1561个问题已完成整改1518个，整改率97%。强力推进“八大工程”。大力实施环境容量管控、大气环境治理等“八大工程”，全市完成工业企业深度治理661家，整治“散乱污”企业2524家。完成“煤改气”“煤改电”、集中供热清洁取暖改造15万户，成功申报国家清洁取暖试点城市，争取到9亿元中央财政资金支持。扎实开展大气专项行动。邀请中国环科院柴发合专家团队、“一市一策”专家团队对我市进行污染源解析和跟踪服务，先后制定了打赢蓝天保卫战三年行动计划、大气污染防治年度行动计划、秋冬季大气污染防治攻坚行动方案和PM2.5、SO2等专项治理方案，深入开展了固废大排查、柴油车和散装物料运输车联合执法等10个专项行动。积极实施水土污染防治。率先在全省建立了“河长+巡河员”河道巡查管理体系，整治入河排污口393处。龙祠水源地一级保护区范围内龙祠、晋掌村环境综合整治等项目基本完成，洪洞汾河国家湿地公园通过验收，大力实施涝洰河生态治理工程。新增水土流失治理面积65.9万亩，完成营造林49.7万亩，全市森林覆盖率达31.4%，三北防护林体系建设工程被评为全国先进。89家煤矿企业启动矿山生态环境恢复治理，全面筑牢了生态保护屏障。

　　(五)抓扶贫、打硬仗，着力提升脱贫质量。深入贯彻中央、省的脱贫攻坚决策部署，紧紧围绕全市“1351”脱贫攻坚思路，下大力气推进精准扶贫、精准脱贫。健全完善脱贫工作体系。创新构建了脱贫攻坚目标责任和工作落实两个体系，在全市实行了“周例会月报告季总结”和“五清单两标准一档案”制度，制定了《坚决打赢全市脱贫攻坚战三年行动的实施意见》，专门派出驻县工作组进行跟踪指导、巡回督导，确保责任到人、措施到位。加快实施十大扶贫工程。市县两级投入扶贫资金6.1亿元，全力推进“十大扶贫工程”。特色产业扶贫“8311”重点项目超额完成省定任务。易地扶贫搬迁7个贫困县57个集中安置点主体竣工。光伏扶贫新建的332座村级光伏电站全部建成并网。10个贫困县建档立卡贫困户完成退耕还林4.2万亩。贫困村提升、健康扶贫、金融扶贫等工程也取得了明显成效。集中攻坚深度贫困。聚焦深度贫困，在政策举措、资金投入、工程项目、帮扶力量等方面，加大向大宁、永和两个省级深度贫困县倾斜力度，强力推动“10+1”共享政策和专享政策落地见效。“五联五帮”责任制持续深入，形成了攻坚深度贫困的工作合力。大宁县“村社一体”改革推动脱贫攻坚的做法得到省委、省政府肯定，蒲县“道德银行”扶贫扶志模式成为全省典型，黎掌村两次登上央视新闻联播。

　　(六)抓安全，控风险，全力确保社会稳定。牢固树立总体安全观，按照“三个坚决防止”和“四铁”要求，防范化解重大风险隐患，全力加强安全生产、社会治理等工作。在防控重大风险上，加大政府债务和金融风险防控力度，把全部债务纳入地方全口径债务监测平台系统，实行网上监控，严厉打击恶意逃废金融企业债务和非法集资行为，市县两级政府债务规模均在可控范围之内。在狠抓安全生产上，制定了《贯彻落实〈地方党政领导干部安全生产责任制规定〉实施细则》，切实强化“五化一杜绝”，深入开展安全生产执法年活动，扎实推进安全生产大检查和专项整治，严肃查处生产安全事故，工矿企业连续9年未发生重大事故，其中煤矿企业连续6年未发生较大以上事故，百万吨死亡率控制在省控指标以内。在维护社会稳定上，强力开展扫黑除恶专项斗争，打掉黑恶势力犯罪团伙110个，破获各类刑事案件815起。持续完善社会治安防控体系，扎实推进“平安临汾”“法治临汾”建设，全市社会大局和谐稳定，为经济社会发展提供了坚强保障。

　　(七)抓民生、提品质，努力增进民生福祉。民生支出累计执行320.7亿元，占一般公共预算支出的83.1%，比上年增长19.3%，人民群众幸福感、获得感、安全感不断增强。文化方面，市博物馆建成开馆，市图书馆、国学馆、射击飞碟靶场等项目顺利推进，县级“四馆一场一院”建设取得积极成效，古县图书馆荣获“国家一级图书馆”，公共文化设施建设不断完善。“平阳记忆”“印象临汾”工程成果显著。“尧王杯”系列赛事圆满举办，竞技体育在亚运会上实现金牌“零”的突破。教育方面，全市认定普惠性民办幼儿园68所。市区新(改、迁)建的8所中小学(幼儿园)进展顺利。尧都区通过国家义务教育均衡验收。完成中小学薄弱校改造156所。高考实现达线人数、达线率、清北人数“三个历史性突破”。立德树人、“三优”评选以及校长、教师交流等工作扎实开展。卫生健康方面，医疗医药医保联动改革扎实推进，109个病种实现按病种收(付)费。永和、曲沃、安泽、古县成功创建县乡医疗卫生机构一体化改革“省级示范县”。医联体建设实现所有县(市、区)全覆盖。市中心医院、市妇幼保健院顺利通过“三甲”复审。建成16家中医馆，中医药服务能力稳步提升。3个县、5个乡镇创建国家卫生城镇工作取得积极进展。

　　(八)抓班子、强队伍，高效推进政府建设。依法接受人大监督，自觉接受政协民主监督，主动接受社会和舆论监督，全年共办理全国政协提案2件，省人大代表建议11件、省政协提案2件，市人大代表建议156件、市政协提案242件、意见11件，办结率达100%。深入开展“强化责任、严守纪律、树好形象”专项治理活动，严格落实中央“八项规定”精神，坚决反对形式主义、官僚主义，驰而不息纠正“四风”。全面压实从严治党主体责任，认真履行“一岗双责”，修订了《临汾市人民政府党组关于落实意识形态工作责任的实施细则》，全年开展集体约谈、提醒谈话和诫勉谈话800余人次，党员干部“四个意识”“四个自信”“两个维护”显著增强，保持了良好的政治生态和干事创业氛围。

　　与此同时，国防动员、双拥共建和优抚安置工作扎实推进。外事侨务、残疾人、红十字会、妇女儿童等事业取得新进步，人防、统计、审计、气象、住房公积金等工作取得新进展，对口援疆工作取得新成绩。

　　各位代表，过去一年的奋斗凝聚艰辛，过去一年的成绩来之不易。这是坚定不移贯彻落实习近平新时代中国特色社会主义思想的结果，是省委、省政府和市委正确领导的结果，是市人大、市政协监督支持的结果，是全市上下勠力同心、拼搏奉献的结果。在此，我代表市政府，向全市人民，向人大代表、政协委员、各民主党派、工商联、无党派人士和人民团体，向驻临部队、武警官兵和所有关心支持临汾发展的各界朋友，表示崇高的敬意和衷心的感谢!

　　安不忘危，兴不忘忧。在肯定成绩的同时，我们也清醒地认识到，全市发展绝非一路坦途，前进的道路上，依然面临着不少困难和问题。主要是：产业结构单一。煤焦冶电四大传统产业占比仍高达90.9%，除装备制造业外，其余新兴产业和“N”个前沿产业推进都不够快。发展动能不足。项目储备不优，落地、开工不足，大项目好项目少;工业经济新旧动能转换不快，新产业、新业态、新模式依然较少，支撑经济发展的动能不足。环境问题突出。我市空气质量综合指数等指标在全国、全省和汾渭平原排名仍处在后位，全市环境基础能力建设相对滞后，能源消耗和排放仍然很大，节能减排任务艰巨。基础设施薄弱。水、电、路、网建设不同程度地存在“短板”，特别是全市农村公路技术等级普遍偏低，人均水资源占有量仍低于全省、全国均值。发展环境不优。服务项目、企业的政策措施落实还不到位，并联审批机制有待优化，开放平台支撑比较弱，营商环境、引智环境仍需改善，等等。这些问题与新时代发展的要求、与群众日益增长的对美好生活的向往还不相适应。对此，我们将直面问题，勇于担当，尽心竭力加以解决。

　　各位代表，临汾的发展正处在一个大调整、大转型交织并存的历史关头，正处在一个大挑战、大机遇相互叠加的关键路口。当此进则全面振兴、退则必然落后的关键时刻，唯有致力改革创新、奋发有为，才能实现全市工作更大的突破。我们要按照市委提出的“必须坚持工作主线、必须牢牢把握总基调、必须促进区域协调发展、必须加强预期引导”的“四个必须”要求，着力解决好“思想理念、经济布局、产业结构、发展环境、市场活力”等事关临汾发展的“五个方面”重大难题，强力促改，主动求新，深入谋转，勇于任事，推动全市经济社会发展取得新成效。

　　二、2019年工作安排

　　2019年是新中国成立70周年，是决胜全面建成小康社会、实现第一个百年奋斗目标的关键一年，也是我市经济转型发展的关键一年。今年政府工作的总体要求是：以习近平新时代中国特色社会主义思想为指导，全面贯彻党的十九大和十九届二中、三中全会精神，深入贯彻习近平总书记视察山西重要讲话精神，按照“巩固、增强、提升、畅通”八字方针要求，在市委的坚强领导下，坚持稳中求进总基调，坚持新发展理念，坚持高质量发展，坚持把深化供给侧结构性改革与深化转型综改区建设相结合作为经济工作的主线，坚持深化市场化改革，扩大高水平开放，继续打好三大攻坚战，统筹推进稳增长、促改革、调结构、惠民生、防风险工作，进一步稳就业、稳金融、稳外贸、稳外资、稳投资、稳预期，紧紧围绕“示范区”“排头兵”“新高地”三大目标，深入推进“345”发展战略，坚定信心，排难而进，努力打造新优势、培育新动能、树立新形象，争当全省转型发展“排头兵”，推动从严治党向纵深发展，激励各级干部改革创新、奋发有为，为全面建成小康社会收官打下决定性基础，以优异成绩迎接中华人民共和国成立70周年。

　　主要预期指标是：地区生产总值增长6.3%左右，全社会固定资产投资增长10%左右，社会消费品零售总额增长7.5%左右，一般公共预算收入增长6.3%以上，城乡居民人均可支配收入分别增长6.5%和6.5%以上，城镇新增就业47000人，居民消费价格涨幅控制在3%左右，城镇调查失业率、城镇登记失业率分别控制在6.5%、4.2%以内。资源节约、环境质量、民生改善等约束性指标完成省定目标。围绕以上目标，今年重点抓好以下十项工作：

　　(一)在产业转型上狠下功夫，推动高质量发展取得新突破

　　坚持区域协调高质量发展战略，强力推进百里汾河、百里黄河、百里太岳“三个百里”经济带建设，把构建“4+6+N”现代工业产业体系作为主攻方向，持续优化调整产业结构，争当能源革命“排头兵”，不断提升我市经济发展的“含金量”“含新量”“含绿量”。

　　强力推进“三个百里”建设。坚持把“三个百里”作为产业转型“主战场”。百里汾河经济带“腾笼换鸟”。推动传统产业优化升级，逐步转移，腾出转型发展空间。依托临汾、侯马陆港(侯曲)、洪洞、襄汾、霍州等经济技术开发区和尧都高新技术产业开发区，按照不同的功能定位，因地制宜，突出特色，承接国外和沿海发达地区产业转移，加快发展现代高效农业，培育壮大战略性新兴产业，优先发展高端服务业，建设资源型经济转型发展创新高地。百里黄河经济带“双轮驱动”。充分发挥国家重点生态功能区优势，坚持生态优先、尊重自然，依托丰富旅游资源，大力发展“文旅+农业”，依托优良自然禀赋，大力发展“传统产业+新能源”。围绕西山7县，整体创建沿黄现代农业文化旅游开发区，打造黄河经济带新的增长极。百里太岳经济带“多点突破”。在生态优先前提下，积极承接百里汾河经济带及区域外产业转移。依托安泽、翼城经济技术开发区和古县涧河、浮山中强等工业园区，建设工业转型升级和资源循环发展利用示范基地。依托太岳山区生态环境优势，培育壮大现代特色农业，大力发展生态休闲旅游。

　　加快改造传统产业。推动传统产业在“长高”上下功夫，坚持走“减、优、绿”之路，通过高新技术注入，脱胎换骨，提档升级，强力实施煤炭、焦化、钢铁等行业转型升级三年行动计划，实现以旧换新，老树发新枝。煤炭产业，坚定不移提高先进煤炭产能比重，持续走深加工、清洁高效利用之路，培育壮大延伸煤炭绿色转化产业链条，增加煤炭的附加值，全市生产煤矿全部达到二级以上安全生产标准。焦化产业，着力提高焦化产业集中度，上大关小，减量置换，退城入园，实现焦化产业优化升级。稳步推进大机焦建设，延伸焦化下游化产深加工产业链，重点推进安泽蔺鑫30万吨/年(5亿立方/年)LNG清洁能源、同世达SNG清洁能源等项目。钢铁产业，以减量置换升级为主要途径，推动存量产能向优势企业集中，走集约型、集团化之路。推进晋南钢铁集团建设，实施曲沃通才产能减量置换项目，加快打造精品钢基地。电力产业，持续优化调整电源结构，积极发展风电、水电以及太阳能等新能源，重点推进吉县远景、隰县盾安、乡宁中电投等一批风力发电项目。

　　大力培育新兴和前沿产业。推动新兴和前沿产业在“长大”上下功夫，坚持做“扶、引、育”文章，积极培育一批新的优势产业聚木成林，实现集群化、规模化、高端化发展。重点发展高端装备制造业。充分利用我市精密铸造、锻造等产业基础，大力扶持一批铸造、装备制造、智能制造企业发展，加快建设“制造强市”，重点打造5个铸(锻)造基地。以华翔为依托，打造以“装备制造+信息化自动化”为特色的中美国际产能合作产业园。在翼城县和侯马市分别打造以“装备制造+军民融合”为特色的翼城高端铸(锻)造军民融合产业园和侯马汽配产业基地。充分发挥临汾职业技术学院教科研优势，在尧都区打造以“装备制造+产学研”为特色的中韩国际产能合作产业园。在襄汾县整合现有铸造企业，打造以“集约化、集团化”为特色的绿色铸造园区。重点推进华翔3D打印产业化和V法自动化改造、汤荣双金属复合一体式制动毂改造等项目建设。大力推进新能源、新材料产业发展。重点依托我市丰富的煤层气资源，加大开发力度，发展关联性产业，加快推进永和丰百能源LNG液化调峰站、大宁鸿晋一次性防护手套二期项目建设。加快打造新材料产业集群，重点推进汾西其亚氧化铝及高精铝板带箔、侯马北铜铜精矿处理、浮山嘉鑫4万吨超高功率石墨电极等项目建设。支持乡宁紫砂小镇(紫砂紫陶产业园区)建设。瞄准国家先进战略，发展大数据、物联网产业，重点推进大数据产业园、飞虹微纳米、光宇LED等项目建设。

　　大力发展民营经济。坚持“两个毫不动摇”，深入贯彻习总书记在民营企业座谈会上提出的大力支持民营企业发展壮大6个方面政策举措，认真落实省委、省政府支持民营经济发展30条意见，着力破解民营经济发展中的问题。扎实开展“政策兑现活动”，持续加大财税、信贷、融资、用地、人才等方面支持力度。利用市产业引导发展基金，引导和带动社会资金支持初创期中小微企业创业创新。完善领导干部联系民营企业制度，健全企业家参与涉企政策制定机制，构建“亲”“清”政商关系。扩大市级企业应急周转资金规模，撬动引导社会资本解决融资难题，鼓励民营担保公司参与支持小微企业发展。积极引导推动民企上市，加快推进华翔集团在主板上市，鼓励支持企业在山西股权交易中心挂牌交易，争取在“新三板”上市。加快推进民营企业集群发展，采取并购、战略联盟、相互持股等方式实现强强联合，着力解决民营企业产业规模小、布局散、层次低等问题。

　　健全完善创新体系。坚持把创新摆在核心位置，加快推进高新技术产业园区建设，新增高新技术企业5家。推动产学研深度融合，加快推进与中科院、中国人民大学、太原理工大学、山西师范大学等院校合作项目落地，培育省级及以上重点实验室和工程技术研究中心5家，科技企业孵化器2家，众创空间12家。全面提升中小微企业核心竞争力，新培育小微企业5000户。持续用好“市长创新奖”，积极推广华翔集团“双创”经验，大力培育“平阳工匠”，将科技成果转化为现实生产力。健全完善人才引进机制，积极引进一批适合临汾民企发展的尖端创新人才和高层次创新团队，培育100名科技型领军人才、500名创新型技术人才、2000名技能型实用人才。

　　各位代表，临汾经济转型，已经“到了一个船到中流浪更急、人到半山路更陡的时候，到了一个愈进愈难、愈进愈险而又不进则退、非进不可的时候”。我们要始终保持“咬定青山不放松”的坚韧意志和战略定力，下定决心“腾笼换鸟”，下大力气转换动能，下足功夫做强支撑，全力打造临汾产业转型“升级版”，努力争当全省转型发展“排头兵”!

　　(二)在激发活力上狠下功夫，推动改革开放开创新局面

　　改革关乎国运，创新决胜未来。我们要坚定不移地用好改革开放“关键一招”，全力破解影响制约发展的体制机制障碍，拓展发展空间，厚植发展优势，全力构建对外开放“新高地”。

　　扎实推进供给侧结构性改革。巩固“三去一降一补”成果，重点在破、立、降上下功夫。大力破除无效供给，化解过剩产能，引导煤炭、焦化、钢铁企业加强技术改造、加快优化升级，实现创新、绿色、集聚发展。全面落实各项税收优惠政策，持续清理规范涉企收费，打好降成本“组合拳”，让企业卸掉重负、轻装上阵。加快建立多主体供应、多渠道保障、租购并举的住房制度，因城因地去库存，优化供需结构，促进房地产业持续健康发展。

　　加快推进开发区改革创新。坚持把开发区作为转型综改“示范区”建设的主阵地，加快临汾侯马综合保税区设立及建设，推动临汾经济技术开发区升级，争取早日获批国家级经济技术开发区。加快完成临汾、侯马、洪洞、襄汾、安泽5个经济技术开发区总体规划、控制性详细规划和规划环评报告，加快推进侯马、安泽、襄汾经济技术开发区扩区步伐，高标准推进起步区建设。加快霍州、翼城、汾西和沿黄现代农业文化旅游开发区的申报和设立。深入推进开发区“三化”改革，积极创建中外合作园区。

　　强力推进重点领域改革。国企国资方面，制定市属企业处置“僵尸企业”工作方案，通过兼并重组、债务重组、清理淘汰等方式，全面推进企业“处僵治困”，力争完成“三供一业”维修改造。营商环境方面，全面深化“一门一网一次”改革，推行“一窗受理、集成服务”模式，建设覆盖全市、联通全省的一体化服务平台。加快市场监管改革，全面推行市场主体注册登记全程电子化和电子营业执照应用，推进“双随机、一公开”监管统一化、常态化。深化商事制度改革，持续推进“证照分离”，降低准入制度性成本。建立免费帮办、银企代办等服务机制，为市场主体提供多渠道高效便捷服务。财税体制方面，加快推进省以下财政事权和支出责任划分改革，争取在基本公共服务领域取得突破性进展。深入推进预算、资产、绩效等管理改革，使财政资金花得其所、用得安全。质量标准方面，大力实施质量强市战略，全面加强质量监管，建立标准化体系，提升质量标准。统筹推进金融体制、集体林权制度配套、电力体制等改革，推动全市各项改革实现新突破。

　　大力提升对外开放水平。深度融入“一带一路”、京津冀、环渤海、黄河金三角、关中平原城市群等区域交流合作。积极组织企业参加第二届中国国际进口博览会、中国国际贸易洽谈会等国内涉外展会，着力开拓国际市场。加快与澳大利亚杰拉尔顿市、美国核桃市的友好城市建设。加快培育跨境电商和外贸综合服务企业，建设市级跨境电子商务公共服务平台，认定一批跨境电子商务示范园区、示范企业和公共海外仓。加快推进中欧班列市场化运作、常态化开行，构建更高层次对外开放新格局。

　　各位代表，我们要把改革开放的旗帜举得更高更稳，以“永远在路上”的精神，一棒接着一棒跑下去，让临汾改革开放的大门越开越大，让临汾改革开放的道路越走越宽!

　　(三)在污染防治上狠下功夫，推动生态治理取得新进展

　　牢固树立“绿水青山就是金山银山”的发展理念，紧紧围绕环境质量改善目标，坚持治标治本并重，强力推进“八大工程”，统筹推进气、水、土治理，加快建设天蓝地绿、山水相融的美丽临汾。

　　突出大气污染防治。实施优化产业结构工程。着力破解我市“一煤独大”的产业结构问题，持续优化产业布局特别是工业布局，严格环境准入，加快推进工业企业转型升级，深入推进重点行业深度减排，持续开展“散乱污”企业整治，不断强化工业无组织治理，提高污染防治水平。实施优化能源结构工程。千方百计降低煤炭消耗总量，加大散煤管控力度，持续开展燃煤锅炉整治，紧紧抓住我市入选北方地区冬季清洁取暖试点城市的机遇，持续扩大集中供热、“煤改电”“煤改气”范围，按计划完成清洁取暖改造任务。实施优化交通结构工程。加快推进车辆结构升级，加强车用油品监管，鼓励推广使用新能源汽车，积极推进清洁运输，不断强化机动车和非道路移动机械污染防治，加强常态监管，形成长效机制。实施优化用地结构工程。优化产业用地、城市建设用地结构，加快城市绿道绿廊建设，提高城市建成区绿化覆盖率。加大扬尘源头治理，严格建筑工地管理，加强生活源管控力度。

　　统筹水土污染治理。打好“碧水保卫战”。深入实施“水十条”，全面落实河长制，加快修复河流生态系统，加强饮用水源地环境保护，强化县、乡两级水源地规范化建设和入河排污口管理。加强工业废水深度处理，大力推进工业企业中水回用工程建设。全面完成污水处理厂提质改造和保温提效工程，确保出水稳定达地表水Ⅴ类标准。打响“净土保卫战”。大力实施“土十条”，全面推进固体废物整治，扎实开展危险废物规范化管理年活动，加快推进生活垃圾焚烧发电厂、餐厨垃圾处理厂建设。建立健全农村垃圾村收集、镇(乡)转运、县处理体系，完成非正规垃圾堆放点80%的整治工作。

　　加强生态保护修复。全面提升国土绿化质量和水平，大力推进西部生态脆弱区、东部水源涵养区、中部百里汾河经济带湿地植被恢复区三大生态屏障建设，完成营造林34.2万亩。加快推进煤炭开采企业矿山生态环境恢复治理工作，全面启动灭失主体矸石山治理，严肃查处煤炭企业矸石违法处置、违法倾倒等破坏生态问题。续建实施汾河流域生态修复尧都区吴村段、侯马市浍河生态修复综合治理等工程。申报实施乡宁县马壁峪、翼城县浍河、吉县清水河、洪洞县洪安涧河、汾西县团柏河和蒲县昕水河等6个中小河流治理项目。推进实施固沟保塬、淤地坝除险加固、小流域综合治理等水土保持项目。

　　提升环境监管水平。启动生态保护红线、环境质量底线、资源利用上限、环境准入清单编制。加快推进环境基础能力建设，建立市县乡三级生态环境监测监控体系，完善环境空气质量监测网络，启动污染防治信息化指挥中心建设，完成机动车尾气遥感监测、河流自动监测等系统建设。强化跨区域、跨部门联合执法，加大环境违法行为依法查处力度，保持铁腕治污的高压态势。充实基层环保工作队伍，加强生态环保工作网格化管理，实现环境监管全覆盖。

　　各位代表，“踏平坎坷成大道，斗罢艰险又出发”。我们要继续以最大的决心、最严的制度、最强的举措，坚定信心，保持定力，打赢污染防治攻坚战，不唤回临汾的蓝天碧水，决不收兵!

　　(四)在精准施策上狠下功夫，推动脱贫攻坚夺取新胜利

　　坚持精准扶贫精准脱贫基本方略，强化目标导向、问题导向、严实导向，夯实脱贫基础，聚焦深度贫困，推进十大工程，实现脱贫攻坚决战决胜。

　　全面夯实脱贫基础。健全完善脱贫攻坚目标责任和工作落实“两大体系”，持续强化动态、精细、台账“三个管理”，引深推行“五清单两标准一档案”“周例会月报告季总结”制度，夯实精准扶贫基础。坚持问题导向，对中央巡视、国考、省考等发现的问题进行“大起底”“回头看”，建立整改台账，坚决整改到位。深化脱贫攻坚作风建设年活动，引深“四级书记遍访贫困”活动，继续实施驻村帮扶“六大行动”，持续提升广大群众的获得感和满意度。强化2020年“交总账”意识，按照摘帽子不摘责任、不摘政策、不摘帮扶、不摘监管“四不摘”要求，加强已“摘帽”贫困县后续政策配套，做好非贫困县基础工作，建立返贫预警机制，巩固脱贫成果。

　　集中攻坚深度贫困。全面落实《临汾市聚焦深度贫困集中力量攻坚三年行动计划(2017—2019)》，坚持用足用好用活省委攻坚深度贫困“10+1”政策，在资金、项目等方面集中向永和、大宁、汾西3个贫困县倾斜，重点做好脱贫“摘帽”基础工作，逐县研判，逐项对标，逐个突破。确保永和、大宁、汾西3个贫困县“摘帽”，81个贫困村退出，2万名建档立卡贫困人口脱贫。

　　持续推进“十大工程”。特色产业扶贫工程。大力构建“1+X”产业扶贫新格局，推动干鲜果、畜牧、蔬菜等特色产业规模化、集约化、品牌化、效益化发展，巩固提升“五有”成果。培育引进一批实力强、规模大的新型经营主体，不断完善新型经营主体与贫困户利益联结机制。推进电子商务进农村综合示范工作，加快推进“快递下乡”，完善乡村物流配送体系，实现全市151个乡镇快递网点全覆盖。按照“县建县管”原则，强化光伏电站运维管理，管好用好收益资金。易地扶贫搬迁工程。完成大宁、永和、汾西3县9087人易地扶贫搬迁任务，77个深度贫困自然村实现整体搬迁。大力推进拆旧复垦工作，跟进产业就业帮扶，激发搬迁户内生动力，确保“搬得出、稳得住、逐步能致富”。生态扶贫工程。持续实施“五个一批”林业生态建设扶贫工程，扩大退耕还林范围，深化林地流转、资产收益改革，规范发展造林合作社，落实选用贫困群众担任护林员等制度，实现生态建设与脱贫攻坚“互促双赢”。贫困村提升工程。以永和、大宁、汾西3个县贫困村为重点，加快补齐水电路网等基础设施和学校、医疗、文化等公共服务“短板”。推进已退出贫困村基础设施和公共服务提档升级。健康扶贫工程。全面落实“三保险三救助”、医保目录内住院医疗费用“136”报销、先诊疗后付费“一站式”服务等政策，确保城乡居民基本医保100%覆盖农村贫困人口，贫困人口就医自付比例控制在10%以内。社会保障政策落实工程。继续精准落实社会保障4方面24项政策，按照“一家一案”制定家庭经济特殊困难学生扶贫方案。继续推动农村低保和扶贫开发“两线”衔接，将符合条件的老年人、残疾人、重病患者、丧失劳动能力人口以及因学致贫、因病致贫等人口纳入低保，落实好重度残疾人护理补贴发放工作。消费扶贫工程。研究制定我市开展扶贫消费的指导意见，开展“百千万”消费扶贫大行动，建立“五进九销”活动长效机制，组织引导本地区农产品批发市场、商贸流通企业和机关、学校、医院、企事业单位等与贫困地区建立长期稳定的供销关系。金融扶贫工程。加大金融部门扶贫贷款力度，创新产业扶贫信贷产品和模式，支持保险机构在贫困地区开发和推广特色农业险种，开展扶贫小额贷款保证保险等业务，着力解决贫困农户贷款难题。扶志扶智工程。强化政策引导、教育引导、典型引导，持续开展“孝亲敬老”“扶贫公益专岗”“倡导新风”等六大行动，推广蒲县“道德银行”先进做法，有效激发贫困群众的内生动力。社会力量帮扶工程。加大企业帮扶、商会帮扶、协会帮扶、结对帮扶力度，继续发挥共青团、妇联、工会、工商联、慈善协会等群团组织和社会组织的桥梁作用，深化“双心双实”、民营企业“百企帮百村”精准扶贫行动和“三晋巾帼脱贫行动”。

　　各位代表，脱贫攻坚时不我待，发起总攻在此一举。我们要以贫困不除愧对历史、小康不达誓不罢休的政治担当，集全市之力向深度贫困地区“宣战”，确保实现全市所有贫困县脱贫“摘帽”，坚决打赢这场脱贫攻坚战!

　　(五)在积蓄后劲上狠下功夫，推动项目建设迈出新步伐

　　发挥有效投资对经济增长的推动作用，扎实开展“深化转型项目建设年”活动，以项目带动争取转型主动，以精准引进实现发展快进，以优质服务保障建设任务，努力为经济转型提供强有力支撑。

　　扩大项目投资。充实完善项目储备库，实现年度“项目储备库总投资达到6000亿元以上”目标，为固定资产投资稳步增长提供不竭动力。紧抓国家加大基础设施领域补短板力度、国家支持民营经济发展、开发区改革创新等机遇，加强与国家和省直部门对接，引导鼓励民企积极投资，在产业转型、基础设施、生态环保、社会民生等领域，谋划实施一批打基础、利长远、补短板、增动能的重点项目，实现产业接链补环，增强转型发展后劲。今年动态监测产业转型、基础设施、社会民生3大类560个项目，总投资2400亿元，年度计划投资600亿元。

　　完善推进机制。健全完善领导包联、项目库择优遴选动态调整、督办考核等机制，构建环环相扣、有序衔接、动态管理的项目推进体系，全力保障项目组织实施。不断强化重点工程调度机制，干部下基层到一线，帮助项目单位解决“难点”“堵点”“痛点”问题，确保重点项目、重点工程顺利推进。推行“承诺制+并联审批”模式，推进“承诺制+标准地”改革，实施“区域评估”，试行“告知承诺制”和“容缺受理”制度，大幅压缩核准类项目审批评估时限，加快项目前期手续办理。

　　加强要素保障。强化土地保障，严格土地利用总体规划管理，重点做好“整案制”，加快推进批而未供和闲置土地清理工作，切实提高土地使用效益。强化资金保障，按照“保重点”的原则，强化项目资金筹措，积极探索投融资新模式，多渠道解决项目融资难题。强化基础保障，做好项目水、电、路、网等“九通一平”工作，破除制约项目开工建设的实质性障碍，为项目早开工、快推进提供有力保障。

　　实施精准招商。健全完善产业链招商指导目录，瞄准京津冀、长三角一体化和粤港澳大湾区等产业发展优势区域，确定重点招商对象，采取小分队、专业化等方式，引导外来投资向战略性新兴产业、现代服务业等领域倾斜，开展精准招商。发挥开发区引领作用，以临汾、侯马经济技术开发区为主，精心组织大型综合性招商推介活动，加强与国内500强和行业50强领军企业对接合作，引进一批能提升产业链、创新链、价值链的优质项目。充分发挥工商联和商会积极引导作用，加强与临汾籍在外企业家沟通交流，激发临汾在外企业家的投资热情，推动晋商晋才回乡创业创新。

　　(六)在乡村振兴上狠下功夫，推动“三农”工作实现新发展

　　坚持以《乡村振兴战略总体规划(2018-2020)》为总抓手，按照产业兴旺、生态宜居、乡风文明、治理有效、生活富裕的要求，统筹推进“五个振兴”，推动农业提质增效、农村文明进步、农民增收致富。

　　做强特色现代农业。围绕发展现代农业，大力实施粮食安全、杂粮振兴、果业提质、中药崛起、绿色蔬菜“五大工程”，全年粮食播种面积稳定在750万亩以上，粮食产量保持在20亿公斤以上;杂粮播种面积稳定在95万亩以上;新发展和改造老果园6万亩，加快创建一批标准化果园;新发展中药材6万亩;新(改)建设施蔬菜0.3万亩，开发露地特色蔬菜2万亩。开展农产品深加工提升行动，推进养生保健、功能食品开发，培育储备一批国家级、省级、市级农产品加工龙头企业，示范创建一批产业化联合体，力争农产品加工销售收入达到110亿元以上。实施农业标准化战略，加快推进“三品一标”认证，积极申报创建“吉县苹果”“隰县玉露香梨”国家级现代农业产业园和“安泽连翘”国家级特优区。各县(市、区)依托产业优势创建“1+N”个区域公用品牌。加快推进耕地质量提升、农水集约增效、旱作良种攻关、农技集成创新、农机配套融合和绿色循环发展等建设，创建5个省级、10个市级“有机旱作农业封闭示范片”。加强非洲猪瘟防控工作，确保生猪产业健康发展。

　　加快发展新兴业态。以尧都、侯马、霍州“一区二市”为重点，辐射带动曲沃、襄汾、洪洞等县，打造百里汾河三产融合发展带，创新流通方式和流通业态，推进鲜活农产品冷链物流，提高互联物联信息技术水平。重点推进尧都城郊农业示范县(区)、襄汾农林文旅康养试点县、曲沃农业产业强镇建设。推动农业与旅游、教育、文化、康养等产业深度融合，打造一批农业主题公园、休闲观光采摘园、特色风情小镇、田园综合体和高端民宿聚集区，不断丰富“一小时经济圈”内涵。

　　改善农村人居环境。学习借鉴浙江“千村示范、万村整治”经验做法，深入开展“百村示范、千村整治”活动，推动农村人居环境三年集中整治向纵深拓展，强力推进农村拆违治乱、垃圾治理、污水治理、厕所革命、卫生乡村“五大专项行动”，促进农村基础设施建设提档升级。充分发挥典型引领作用，持续推进美丽宜居乡村创建，按照“四美三宜”标准，因地制宜打造不同类型、不同特点、不同模式的美丽宜居乡村，推进乡村特色化、景区化、规范化、标准化建设。继续清理整治“大棚房”，坚决遏制“农地非农化”。

　　深化农业农村改革。推进农村改革再出发、再深化，突出抓好农村基本经营制度、农村土地制度、农村集体产权制度、农业支持保护制度、农业科技体制、乡村治理体系、脱贫攻坚与乡村振兴衔接机制、有机旱作农业和城郊农业发展体制等8项重点改革任务，分门别类在县乡村三级试点推行，典型引路，全面铺开。着力构建农村新型经营体系，围绕农民新型化、专业化、职业化的发展趋势，大力培育专业大户、家庭农场、专业合作社等新型主体。着力构建农业社会化服务体系，积极推行统防统治、土地托管、代耕代种、机种机收等服务模式。着力构建乡村振兴人才体系，大规模、高质量培育新型职业农民。

　　各位代表，农业兴，天下安。临汾有着得天独厚的农业资源，在推进乡村振兴的道路上大有可为，理应走在前列。我们要加快农业结构调整、体制优化、产业升级，努力打造农业强、农村美、农民富的乡村振兴“临汾样板”!

　　(七)在文旅融合上狠下功夫，推动三产发展实现新提升

　　坚持高端化、生态化、特色化、智慧化发展方向，加快文旅深度融合，大力发展现代商贸物流业，实现第三产业高质量发展，打造推动临汾经济发展的新引擎。

　　加快三大旅游板块建设。以建设国家全域旅游示范区为目标，强力打造根祖、黄河、太行三大旅游板块。加快景区创建工作，推进壶口瀑布、云丘山、尧庙·华门国家5A级景区创建和永和乾坤湾、中镇霍山七里峪等国家4A级景区创建。加快旅游基础设施建设，围绕优化提升“中线根祖文化游、东线太行山水游、西线黄河风情游”3条精品线路，进一步加快旅游道路、厕所、集散中心、咨询服务中心、汽车营地、水电气暖、标识标牌等旅游基础设施建设。持续开展旅游从业人员素质培训，落实好“六字”要诀，强化旅游行业监管，提高景区管理和服务质量。

　　全力推动文旅深度融合。叫响叫亮“中国根·黄河魂”天下华人故里品牌，坚持以开展文化旅游“五个年”活动为抓手，加快推进文旅融合步伐，真正让临汾厚重的历史文化、丰富的文物资源活起来、火起来。统筹推进文化和旅游在发展理念、公共服务、行政审批、市场监管、行政执法、宣传营销等领域全方位对接，大力推动文化市场综合行政执法改革、涉旅文物保护单位“两权分离”改革。高标准制定沿黄现代农业文化旅游开发区总体规划，启动实施沿黄七县全域旅游、特色小镇、田园综合体等重点文旅融合项目，建设具有黄河风情、黄土高原特色的旅游景区，努力打造国际旅游目的地。依托华门光影秀和尧庙·华门景区“两街两馆两中心”，加快实施尧庙·华门文旅融合示范区建设。推进“旅游+”“文化+”，培育发展文化体验游、休闲度假游、康养体育游、自驾房车游、研学游等新业态，推动文旅产品融合、业态融合和产业重构，实现文化和旅游神与形的有机统一、血与脉的水乳交融。

　　大力发展现代商贸服务业。按照新商贸、新物流、新制造的思路，扎实推进侯马市国家服务业综合改革试点工作。加快推进“互联网+物流”发展，加大对侯马方略国际物流港的支持引导，发挥海关优势，提升利用效率。积极整合物流资源，降低物流成本，支持推动兴荣物流、晋南电商物流园区等企业大力发展甩挂运输、多式联运，打造全省交通物流发展示范基地。作为全国首批城乡高效配送试点城市，积极构建高效集约、协同共享、融合开放、绿色环保的城乡配送体系，培育骨干供配企业。深入推进侯马、隰县、大宁等县(市、区)“电子商务进农村综合示范县”建设。全面推广隰县电商模式，着力构建市县乡村四级联创机制。加大消费市场的培育，提升城市消费，扩大乡村消费，推动“工业品下乡，农产品进城”。创新流通方式，培育现代供应链，推动流通企业线上线下融合发展，促进供需有效衔接。发展服务消费，推动家政、餐饮、健康、养老等服务向标准化、便利化、精细化发展。

　　(八)在基础建设上狠下功夫，推动城乡协同发展走出新路径

　　全面贯彻落实以人为核心的城镇化发展理念，实施板块联动，坚持市域统筹，加强基础建设，加快行政区划调整，提高城乡一体化发展质量。

　　坚持规划先行。以先进理念规划中心城市建设，加快推进我市《市域城镇体系规划》《城市总体规划》修编工作。坚持统筹协调、优化布局，尽快编制市区“生态修复、城市修补”规划和清洁供热、垃圾处理、电动汽车充电基础设施等专项规划，为中心城市发展奠定基础。扎实推进区划调整，不断提升我市城市规模、城市承载能力、人口和资源要素吸纳能力，努力把我市打造成“区域中心城市”。

　　提升城市品质。以建设生态宜居城市为目标，坚持“经营城市”理念，大力发展“城市经济”，以“促增长、治环境、补短板”为出发点，围绕“建设新城、改造老城、提升东城、绿色绕城”的发展思路，启动实施35项市区城建项目，当年完成投资45.2亿元。加快推进尧天大街、迎春街南延、体育街南延、汾东立交桥、解放路立交桥等道路建设改造项目，完善城市道路框架;重点推进道路绿化、游园绿化、公园绿化等绿化建设项目，加快国家园林城市创建步伐。积极推进涝洰河片区、万通社区、刘村、段店等21个棚改项目，把城市的“棚户区”打造成城市的“精品区”。加强城市供水、供气、供暖管网改造，不断改善城市人居环境。提高城镇化质量，积极推进农业转移人口市民化，全面放宽落户限制，推动居民证制度全覆盖，实施城镇建设用地增加规模与吸纳农业转移人口落户挂钩机制，提高户籍人口城镇化率。

　　完善基础设施。统筹推进路网、水网、电网、互联网等建设力度，加快补齐基础设施“短板”。加快路网建设。积极创建“全国性综合交通枢纽城市”。开建隰吉高速，完成霍永高速蒲县连接线、长临高速古县连接线等项目，完成沿黄旅游公路100公里。扎实推进临汾民航机场改扩建，加快推进永和、隰县、乡宁、侯马通用机场和瓦日铁路连接线前期工作。以创建“公交都市”为契机，加快全市纯电动公交车更新替换的步伐。加快水网建设。加速大小骨干水网工程建设，重点实施引沁入汾浮山供水工程，力争完工达效。加快引沁入汾尧都专线水质提升工程，推进尧都区仙洞沟、隰县南峪等4座水库建设，启动吉县腰西、汾西申家庄等3座水库立项工作。加快电网和互联网建设。加快推进市区电网升级改造，全面提升供电服务水平。围绕打造“智慧城市”，加快通讯网络建设，提高光纤宽带覆盖率，超前谋划布局5G基站，抢占制高点，推动互联网快速发展。

　　(九)在风险防控上狠下功夫，推动社会治理迈上新台阶

　　牢固树立总体安全观，坚决打好防范化解重大风险攻坚战，高度警惕“黑天鹅”，密切防范“灰犀牛”，重心下移，力量下沉，强化安全生产监管，推进社会治理创新，为全市经济转型发展提供可靠的安全保障。

　　防控各类重大风险。坚持用斗争的精神直面风险，用发展的眼光防范风险，用改革的思维应对风险，用科学的办法化解风险，积极稳妥做好防控风险工作。加强金融风险源头管控，支持银行不良贷款处置化解，加强对影子银行、互联网金融等薄弱环节监管，依法严厉打击各类非法集资行为，促进金融市场平稳健康运行。严控政府债务规模，正确处理好上项目、抓发展与防风险、化债务的关系，本着“量力而行”的原则，坚决遏制隐性债务增量，稳妥处置债务存量，确保债务风险得到有效管控。以严防各类矛盾交织叠加为基点，全力做好矛盾排查调处和风险防范化解工作，完善涉稳因素源头化解和涉稳事端稳控处置机制，坚决防控区域性、系统性风险。

　　守牢安全生产底线。加快推进安全生产领域改革，严格落实地方党政领导干部安全生产责任制，不断健全完善安全生产责任体系，提高安全监管执法效能。深入开展安全生产大检查，深化重点行业领域专项治理，提高安全生产隐患风险防控能力。推进安全生产标准化创建工作，加大安全生产教育培训力度，持续开展“反三违”活动，强化现场管理，提高企业本质安全水平。规范安全监管执法行为，健全常态化暗查暗访机制，强化过程执法，严格落实“四个一律”和“五个一批”要求，依法严厉打非治违。加强应急救援体系建设，推进应急管理信息化，提升各类突发事件应对处置能力，加大防震减灾教育宣传力度，新建市防震减灾科普教育馆，夯实防灾减灾基层基础工作。推动安全红线意识持久化、安全生产标准化、排查治理制度化、监督管理规范化、应急管理信息化、社会监督常态化，有效防范一般事故，切实遏制较大事故，坚决杜绝重特大事故，努力实现事故总量、死亡人数“双下降”。

　　加强和创新社会治理。紧扣新中国成立70周年大庆安保维稳主题主线，部署开展“践行新使命、忠诚保大庆”主题实践活动。严密防范、严厉打击境内外敌对势力各种渗透颠覆破坏活动，坚决捍卫以政权安全、制度安全为核心的国家政治安全。坚决贯彻落实习近平总书记关于扫黑除恶专项斗争的重要指示精神，深入开展扫黑除恶专项斗争，严厉打击各类黑恶势力违法犯罪活动，铲除黑恶势力“保护伞”。持续开展打击文物犯罪，食药、枪爆领域犯罪，盗抢骗、黄赌毒违法犯罪专项行动。加快完善立体化、信息化社会治安防控体系，全面落实重要目标和人员密集场所的安全防范措施，提升社会整体防控能力。巩固加强执法全流程智能管理平台建设，提升规范执法能力水平。大力实施改革强警战略和公安大数据战略，积极构建现代警务体制机制，加快公安大数据应用中心建设，着力打造“数字警务、智慧公安”。

　　各位代表，今年是建国70周年，大事多、要事多、喜事多，防风险、筑平安的任务更加艰巨，必须切实增强责任感和使命感，努力营造安全稳定的良好环境，筑牢防控安全风险的铁壁铜墙!

　　(十)在改善民生上狠下功夫，推动民生事业取得新成果

　　民生无小事，当求细与实。坚持把人民利益摆在至高无上的地位，紧扣人民群众日益增长的美好生活需要，把新增财力优先用于保障和改善民生，多办利民实事，多解民生难事，兜牢民生底线，让改革发展成果更多更公平惠及全市人民群众。

　　优先发展教育事业。贯彻落实全国教育大会精神，巩固义务教育发展基本均衡成果，统筹推进城乡义务教育一体化发展。加快推进洪洞县、霍州市、乡宁县学前教育改革发展省级试点工作，认定普惠性民办幼儿园60所。积极应对新高考改革，支持高中学校多元化发展，实现高考成绩新突破。加大中高等职业院校资源整合，加快推进山西师范大学整体搬迁一期工程，全力推进山西师范大学临汾学院新校区建设。加快推进社区教育工作，努力实现17个县(市、区)社区教育全覆盖。持续深化“三优”评选活动，加强师资队伍和师德师风建设，努力办好人民满意的教育。

　　加快建设“健康临汾”。巩固公立医院改革成果，扩大公立医院薪酬制度改革试点。坚持“三医”联动，统筹推进现代医院管理制度建设和公立医院绩效考核，提升公立医院管理水平。持续深化县乡医疗卫生机构一体化改革，推动医疗卫生机构提质增效。实施人才强卫工程，不断提升医疗卫生、基本公共卫生服务、中医药和妇幼健康服务能力。加强医防、医养融合发展，推动以治病为中心向以人民健康为中心转变。持续推进国家卫生城镇创建工作。

　　大力繁荣文化事业。培育和践行社会主义核心价值观，深入实施公民道德建设工程。全面完成市图书馆、射击飞碟靶场建设并投入使用，启动临汾戏剧研究院非物质文化遗产传承培训基地建设工程。持续实施“平阳记忆”“印象临汾”两大文化工程，打造一批与时代接轨的精品力作，为群众提供更多更好的精神食粮。深入推进文明守望工程、乡村文化记忆工程和文化惠民工程，创新文物保护利用机制。广泛开展全民健身和竞技体育运动，举办马拉松、汾河骑行、五人制足球赛等体育品牌赛事。承办好全国二青会射击飞碟、空手道和花样滑冰项目，举办好全市第五届运动会。

　　稳定就业和拓宽社保。广泛开展岗位练兵、技术比武和职业技能竞赛活动，不断增强“临汾技工”影响力。落实各项补贴政策、开发购买公益性岗位、加大劳务输出、实施兜底援助，持续开展好就业援助专项行动。完善劳动合同和劳动用工备案制度，有效维护和谐劳动关系。巩固全民参保登记计划工作成果，加强社会保障卡建设，稳步提高社会保障待遇水平。加快推进机关事业养老保险制度改革，健全完善工资制度，保障工作人员工资待遇及时兑现。提升城乡社会救助水平，织牢困难群众基本生活安全网。落实好个人收入所得税专项附加扣除等政策。

　　在做好上述工作的同时，还要深入开展双拥共建活动，做好国防动员、退役军人、人民防空、民兵预备役工作;支持工会、妇联、共青团、工商联等人民团体工作;统筹审计、统计、气象、水文、外事、残疾人和关心下一代等各项工作;继续做好对口援疆工作。

　　各位代表，民之所盼，政之所向。我们要始终坚持保基本、兜底线、促均衡、优品质，全面增强人民群众的获得感、幸福感和安全感。今年，市政府将在完成好省政府“八件民生实事”任务的基础上，继续办好“十件民生实事”：

　　一是继续实施《临汾市2018—2020年市区学校建设三年行动计划》，在2018年的基础上，续建、新建或改扩建中小学(幼儿园)13所，解决入学难、入园难问题。

　　二是继续为全市农村放映公益电影3.5万场，为农村寄宿制学校放映爱国主义教育影片3800场，免费送戏下乡1000场，进一步丰富群众文化生活。

　　三是建设市老年康养福利中心，更好满足社会养老服务需求。

　　四是对500名残疾人开展免费职业技能培训，进一步提升残疾人就业能力。

　　五是投资7100万元完成市区36条小街巷的硬化、美化，进一步改善城市环境。

　　六是新(改)建“四好农村路”2500公里，进一步改善群众出行条件。

　　七是为市区公交车配备乘务管理员200名，确保群众乘车安全。

　　八是全面实施新生儿疾病免费筛查3项，进一步提高我市出生人口质量，降低出生人口缺陷。

　　九是继续实施全民技能提升工程，打造“临汾技工”品牌，再培训7万人。

　　十是进一步加大清洁取暖工作力度，完成清洁取暖改造1900万平米，建筑节能提升改造130万平方米，惠及居民5000余户。

　　三、加强政府自身建设

　　人民满意是政府工作的永恒追求。我们要始终坚持党的领导，按照信念过硬、政治过硬、责任过硬、能力过硬、作风过硬的要求，切实加强政府自身建设，全面提升政府治理现代化水平。

　　强化党的领导。坚持把党的领导作为最高政治原则，切实提高政治站位，牢固树立“四个意识”，忠实践行“两个维护”，坚决维护习近平总书记党中央的核心、全党的核心地位，坚决维护党中央权威和集中统一领导，始终做到忠诚核心、拥戴核心、维护核心、看齐核心。坚持以习近平新时代中国特色社会主义思想为指导，深入贯彻落实习近平总书记视察山西重要讲话精神，不折不扣执行省委、省政府和市委的各项决策部署，有力推动各项工作在临汾落地生根。

　　激发创新活力。全面准确把握省委部署和要求，扎实开展“改革创新、奋发有为”大讨论，加强组织领导，周密安排部署，细化工作责任，认真落实好“十个规定动作”，在“六个破除、六个着力、六个坚持”上用实功、见实效。坚持把大讨论与推动经济社会发展紧密结合，增强大讨论的针对性，实现两提高、两促进。总结好、运用好、转化好大讨论的成果，切实提升政府工作的创新力和执行力。广泛宣传“改革创新、奋发有为”大讨论的重大意义，深入挖掘一批好做法、好经验、好典型，在全社会营造改革创新的浓厚氛围。

　　优化政务环境。全面深化、拓展、延伸“13710”工作制度。完成市县乡各级政府和部门的OA协同办公系统应用建设，实现全覆盖。坚持马上就办、真抓实干，大力优化“六最”营商环境，全面深化“一门一网一次”改革，推行“一窗受理、集成服务”模式，重点打造全省“政务服务一张网”示范市，加快建设覆盖市县乡村四级“互联网+政务服务”平台。持续压减和下放行政许可事项，大幅压缩企业开办、水电气报装、获得信贷、不动产登记办理时间。坚决清理群众和企业办事的各类证明，凡是没有法律法规依据的一律取消，让群众办事“最多跑一次”。严格落实公平竞争审查制度，有序清理妨碍统一市场和公平竞争的各类显性和隐性障碍，坚决打破“卷帘门”“玻璃门”“旋转门”。规范政府权力运行，崇尚企业家精神，支持企业家发展，营造公平公正的法治环境、规范守信的市场环境、重商亲商的社会环境。稳步推进机构改革工作，坚持做到队伍不乱、人心不散、工作不断。

　　提升能力本领。加强干部队伍能力建设，持续开展领导干部能力提升讲座，把推动经济高质量转型发展、全面深化改革、扩大对外开放等列入必修课程，不断增强学习、政治领导、改革创新、科学发展、依法行政、群众工作、狠抓落实、驾驭风险等“八个本领”，引导干部克服“本领恐慌”，优化知识结构，拓宽眼界视野，丰富工作思路，努力建设高素质的专业化干部队伍。

　　严格依法行政。加强法治政府建设，严格依法用权、依规履责、依章办事，完善依法决策、科学决策、民主决策机制，推进严格规范公正文明执法，加大行政督查和问责力度。依法接受人大及其常委会法律监督和工作监督，自觉接受人民政协民主监督，主动接受社会和舆论监督，广泛听取人大代表、政协委员、民主党派、工商联、无党派人士和人民团体意见，让权力在阳光下运行。

　　加强廉政建设。始终保持政治定力，严字当头、一严到底，拧紧责任链条，层层传导压力，推动政府系统全面从严治党向纵深发展。严格落实中央“八项规定”精神和省委实施办法，持之以恒克服形式主义和官僚主义，久久为功祛除享乐主义和奢靡之风。深入推进反腐败斗争，坚持无禁区、全覆盖、零容忍，努力营造我市良好的政治生态。

　　各位代表!一切难题，只有在实干中才能破解;一切机遇，只有在实干中才能把握;一切愿景，只有在实干中才能实现。让我们高举习近平新时代中国特色社会主义思想的伟大旗帜，在市委的坚强领导下，在市人大、市政协和社会各界的监督支持下，以坚如磐石的信心、只争朝夕的劲头、坚韧不拔的毅力，心怀梦想，奋勇拼搏，知重负重，敢为善为，奋力推进经济社会高质量发展，为决胜全面小康、实现振兴崛起，在“两转”基础上全面拓展新局面而努力奋斗!以优异成绩迎接中华人民共和国成立70周年!
